# Heightened Plasma Levels of Transforming Growth Factor Beta (TGF-β) and Increased Degree of Systemic Biochemical Perturbation Characterizes Hepatic Steatosis in Overweight Pediatric Patients: A Cross-Sectional Study

**DOI:** 10.3390/nu12061650

**Published:** 2020-06-02

**Authors:** Junaura R. Barretto, Ney Boa-Sorte, Caian L. Vinhaes, Hayna Malta-Santos, Jessica Rebouças-Silva, Camila F. Ramos, Monica A. S. Torres-Nascimento, Valeria M. Borges, Bruno B. Andrade

**Affiliations:** 1 Escola Bahiana de Medicina e Saúde Pública, Salvador 41150-100, Brazil; junaura@gmail.com (J.R.B.); neyboasorte@gmail.com (N.B.-S.); cmilafr.nut@gamil.com (C.F.R.); monicatorres@bahiana.edu.br (M.A.S.T.-N.); 2 Fima Lifshitz Metabolic Unit, Hospital Universitário Professor Edgard Santos, Universidade Federal da Bahia, Salvador 40170-110, Brazil; 3 Departamento de Ciências da Vida, Universidade do Estado da Bahia, Salvador 48000-000, Brazil; 4 Instituto Gonçalo Moniz, Fundação Oswaldo Cruz, Salvador 40296-710, Brazil; caianleal@gmail.com (C.L.V.); haynamalta@gmail.com (H.M.-S.); jeureboucas@gmail.com (J.R.-S.); vborges@bahia.fiocruz.br (V.M.B.); 5 Multinational Organization Network Sponsoring Translational and Epidemiological Research (MONSTER) Initiative, Salvador 41810-710, Brazil; 6 Curso de Medicina, Faculdade de Tecnologia e Ciências, Salvador 45600-080, Brazil; 7 Faculdade de Medicina, Universidade Federal da Bahia, Salvador 40170-110, Brazil; 8 Curso de Medicina, Universidade Salvador (UNIFACS), Laureate Universities, Salvador 41770-235, Brazil

**Keywords:** non-alcoholic fatty liver disease, pediatric obesity, transforming growth factor beta, systemic biochemical perturbation, transaminases, cross-sectional studies

## Abstract

Nonalcoholic Fatty Liver Disease (NAFLD) is a common cause of chronic liver disease in childhood and strongly associated with obesity. Routine biochemical non-invasive tests remain with low accuracy for diagnosis of NAFLD. We performed a cross-sectional study to examine potential associations between anthropometric and biochemical parameters, specially TGF-β, a prognosis marker for hepatic steatosis (HS). Between May and October 2019, seventy-two overweight adolescents were enrolled, of which 36 had hepatic steatosis. Hepatic, lipidic and glycemic profiles, and levels of vitamin D, ferritin and TGF-β were analyzed. Hierarchical cluster and a discriminant model using canonical correlations were employed to depict the overall expression profile of biochemical markers and the biochemical degree of perturbation. Median values of alanine aminotransferase (ALT), gamma glutamyl transpeptidase (GGT), and TGF-β were higher in the adolescents with HS. Values of body mass index (BMI)/age and ALT, but not of TGF-β, were gradually increased proportionally to augmentation of steatosis severity. In a multivariate analysis, TGF-β plasma concentrations were associated with occurrence of hepatic steatosis independent of other covariates. Discriminant analysis confirmed that TGF-β concentrations can identify HS cases. Our data reveal that HS patients exhibit a distinct biosignature of biochemical parameters and imply TGF-β as an important biomarker to evaluate risk of steatosis development.

## 1. Introduction

Non-alcoholic fatty liver disease (NAFLD) represents a generic term that includes hepatic steatosis, nonalcoholic steatohepatitis (NASH), and the evolution to fibrosis or cirrhosis [1,2]. Such a condition develops in the absence of secondary causes, such as medications or metabolic diseases [1,2]. NAFLD is the most common cause of chronic liver disease in adults and children, affecting 2.6% of children and 9.6% of adolescents [3,4,5]. Of note, the prevalence is even higher among obese children and adolescents, varying between 12 to 80% [4,6,7]. Hence, a higher prevalence of childhood obesity and its associated comorbidities, especially metabolic syndrome and dyslipidemia, is followed by increases in the prevalence and severity of NAFLD [1,4,5,8,9,10]. Furthermore, severe obesity is also associated with more adverse clinical outcomes and greater risk of progression to NASH and cirrhosis in childhood [11,12].

In adults, NASH is associated with increased all-cause mortality, cirrhosis, and end-stage liver disease [2,13]. In childhood, despite the lack of data about the NAFLD prognosis in these patients, morbidity and mortality has been described to be higher than in adults [1,5,14]. Despite the fact that there are a number of noninvasive tests and scoring systems available to characterize NAFLD and NASH, the diagnosis of NASH requires a histopathology analysis of liver biopsy demonstrating steatosis, inflammation, and cytological ballooning of liver hepatocytes [2]. Albeit being strictly necessary for confirmatory diagnosis, histopathological examination of hepatic tissue is rarely performed in asymptomatic pediatric patients because it is an invasive method with potential complications [1,15]. In this scenario, biochemical analyzes and ultrasound are the most commonly used methods to perform initial diagnosis of NASH [2]. Of note, the most accessible image examination, standard ultrasound, is able to show the presence of hepatic steatosis, but it lacks accuracy to demonstrate inflammation or fibrosis, which limits its use to identify early manifestations of NASH.

Hepatic steatosis (HS) is necessary to occur but it seems that other mechanisms are required to fully explain the pathogenesis of NASH [16]. Recent studies have shown that the pathogenesis of NAFLD and NASH involves a metabolic perturbation, including impaired glycemic control, altered lipid metabolism, and insulin resistance [17]. In fact, an imbalanced relationship between triglyceride (TG) production or uptake by the liver and clearance or removal is thought to underlie the HS [18]. More recently, it has been suggested that and intricate association between the intestinal microbiome and adipokines secreted by adipose tissue results in lipotoxicity, oxidative stress, mitochondrial dysfunction, and hepatic inflammation [10,18,19].

Several biochemical tests such as alanine aminotransferase (ALT), aspartate aminotransferase (AST), alkaline phosphatase, gamma glutamyl transpeptidase (GGT), insulin, and triglycerides have been studied in the context of diagnostic investigation of NAFLD, but such markers have been shown to exhibit low capacity to clearly identify patients with this condition [1,5,20,21]. Similarly, systemic inflammatory markers, such as ultra-sensitive C reactive protein (US-CRP), which has been previously described as a marker of liver inflammation in adults and children, had proven to be limited to evaluate liver damage [1,5,20,21]. In this setting, novel biomarkers not yet routinely used in clinical practice, such as hemeoxygenase-1 (HO-1), and transforming growth factor beta (TGF-β), have been suggested as having great potential to help assessment of NAFLD [22,23,24,25,26].

Here, we performed a detailed description of biochemical profiles associated with hepatic steatosis in children, to potentially delineate novel insights in the pathogenesis of NAFLD. We also aimed at identifying candidate biomarkers measured in blood that could help diagnose this condition.

## 2. Materials and Methods

### 2.1. Study Design and Participants

We performed a cross-sectional study in Salvador Bahia, northeast of Brazil, with a non-casual sample, including 72 overweight adolescents, screened to a clinical trial and attended at the Bahiana School of Medicine and Public Health outpatient clinic between May and October 2019. Participants of both sexes aged between 10 and 19 years were selected. Overweight, obesity, and severe obesity were defined based, respectively, on the body mass index-for-age (BMI-for-age, z-score) indicator above +1, +2, and +3 z scores using the WHO reference [27]. After signing the informed consent form (legal guardians), all participants were submitted to abdominal ultrasound examination, collection of laboratory tests and medical and nutritional consultation. Patients with obesity of endogenous etiology, had a genetic syndrome, or who had previous liver diseases were excluded from the study.

### 2.2. Nonalcoholic Fatty Liver Disease (NAFLD) Diagnosis

After a minimum period of two hours of fasting, ultrasound examination was performed using a Canon brand Xario 100 Platinum^®^ device, using a 2 to 5 MHz convex transducer after. Hepatic steatosis diagnosis and classification (grades I, II, and III) were investigated, based on changes in liver echogenicity, in the identification of intrahepatic vessels and the diaphragm, according to criteria described by Hamaguchi et al. [28]. All ultrasound examinations were performed at the research institute by a single radiologist (the author M.T.N.).

### 2.3. Clinical, Anthropometric, and Biochemical Procedures

Clinical and anthropometric characteristics were measured by a pediatrician and a dietician, these included weight (kg), height (cm), waist circumferences (cm), and blood pressure (mmHg). A Body Composition Analyzer InBody520^®^ (Biospace Co., Ltd., Urbandale, IA, USA) was used to measure body weight. To measure height, a portable stadiometer^®^ (SECA 206, SECA Medical Measuring Systems and Scales, Hamburg, Germany) was used. The body mass index (BMI) was calculated using the standard formula as kg/m^2^. The abdominal circumference was measured using a non-elastic millimeter tape measure, the reference point being the midpoint between the iliac crest and the last rib [29].

Blood pressure was measured twice using an automatic medical grade monitor OMROM Control HEM-7122^®^ (OMRON Co., Osaka, Japan). The first measurement was taken after five minutes of rest, while participants were sitting with the dominant arm supported at heart level. The second measurement was taken in the same way, five minutes after [30].

An overnight fasting blood sample was collected in a vacutainer from each participant and centrifuged using a standard tabletop centrifuge for the separation of serum using standardized laboratory techniques and sent for assays to determine concentrations of fasting glucose, insulin, total cholesterol (totC), low density cholesterol (LDL-c), high density cholesterol (HDL-c), triglycerides (TG), urea, creatinine, ultrasensitive CRP (us-CRP), aspartate aminotransferase (AST), alanine aminotransferase (ALT), gamma-glutamyl transferase (GGT), alkaline phosphatase, total bilirubin and fractions, vitamin D, free thyroxine (fT4), thyroid-stimulating hormone (TSH) and ferritin using an automated biochemical analyzer (Cobas c8000-Roche Diagnostics International Ltd., Risch-Rotkreuz, Switzerland). In EDTA tubes, hematological analyses were performed using standardized laboratory techniques (XN1000S-Sysmex^®^, Kobe, Japan). Plasma levels of Transforming growth factor β (TGF-β) and heme oxygenase-1 (HO-1) were measured using single enzyme-linked immunosorbent assay (ELISA) (R&D Systems, Minneapolis, MN, USA) according to the manufacturer’s instructions. Of note, we quantified the active form of soluble TGF-β following the manufacturer’s protocol.

### 2.4. Obesity Definition

Abdominal obesity was defined by waist circumference > 90th percentile to sex and to age according to Freedman et al. [29]. Systemic Arterial Hypertension (SAH) was defined according to the American Society of Pediatrics criteria [30]. Diagnosis of Metabolic Syndrome was obtained using the IDF criteria [31]. The severity of hepatic steatosis was categorized on three levels (0 = no steatosis; 1 = mild steatosis-grade I by USG; 2 = moderate/severe-grades II and III by USG).

### 2.5. Statistical Analysis

Descriptive statistics were performed to characterize the study population. Continuous variables were tested for Gaussian distribution using the D’Agostino-Pearson test. No variables exhibited normal distribution. The median values with interquartile ranges were used as measures of central tendency and dispersion, respectively. The Mann-Whitney *U* test (comparing 2 groups) or Kruskal–Wallis test with the Dunn’s multiple-comparison or non-parametric linear trend ad hoc tests (for more than 2 groups) were used to compare continuous variables whereas the Pearson’s chi-square test was used to compare variables displayed as percentages. Hierarchical cluster analyses (Ward’s method) of log10 transformed and z-score normalized data were employed to depict the overall expression profile of indicated biochemical markers in the study subgroups. All comparisons were pre-specified and two-tailed. Differences with *p*-values below 0.05 after Holm-Bonferroni’s adjustment for multiple comparisons were considered statistically significant. Profiles of correlation between biochemical parameters were examined using network analysis of the Spearman correlation matrices. Correlations with *p*-value < 0.05 were included in the network visualization. Spearman rank values (rho) were used to describe the strength of correlations between grade/severity of hepatic steatosis and circulating concentrations of biochemical parameters.

A discriminant model using sparse canonical correlation analysis (CCA) was employed to assess whether a combination of circulating biomarkers could discriminate individuals with hepatic steatosis from those without this condition. Our experience with biomarkers has revealed that not only is the concentrations of a given parameter important, but also the correlation profile with other parameters is critical in evaluation of its involvement in a biological phenomenon [32,33]. Thus, we employed basically two types of analyses: one based on individual concentration values and a second exploring the profile of correlations between several markers in each clinical group. Following this idea, the discriminant model used in the exploration of the data was based on CCA. In this analysis, the way each biomarker correlates with each other, rather than its concentration values, is used to infer differences between the study groups [32,34]. This model is not based on number of correlations, but the quality (whether a correlation is positive or negative) and strength (rho value) are computed to test dissimilarities between the groups.

The molecular degree of perturbation (MDP) was calculated using values of the biochemical parameters to infer the degree of biochemical perturbation (DBP) associated with hepatic steatosis. This method has been used and detailed previously [33,34,35]. In the present study, “No steatosis” was defined as the “reference” group, and the average level and standard deviation of this reference group were calculated for the plasma concentrations of each inflammatory marker. The DBP score of an individual marker in a given sample “s” was defined by taking the difference in concentration level in sample “s” from the average of the marker in reference group divided by the corresponding standard deviation. Thus, the DBP score represents the number of standard deviations from the reference. Individuals who had DBP values above two standard deviations from mean value of controls were considered biochemically perturbed.

A multivariable regression model using variables with univariate *p*-value < 0.2 was performed to assess the odds ratios (OR) and 95% confidence intervals (CIs) of the associations between values of indicated parameters and occurrence of hepatic steatosis. Receiver Operator Characteristics (ROC) curve analysis was employed to test the performance of the plasma levels of TGF-β to distinguish patients with or without hepatic steatosis.

The statistical analyses were performed using GraphPad Prism 8.0 (GraphPad Software, La Jolla, CA, USA), STATA 11 (StataCorp, College Station, TX, USA), JMP 14.0 (SAS, Cary, NC, USA) and R statistical software.

### 2.6. Ethics Statement

The research project was approved by the Ethics and Research Committee of Bahiana School of Medicine and Public Health (protocol 3.095.307/2018). Written informed consent was obtained from all participants or their legally responsible guardians, and all clinical investigations were conducted according to the principles expressed in the Declaration of Helsinki.

## 3. Results

### 3.1. Characteristics of Participants

A total of 72 participants were enrolled, 36 without hepatic steatosis and 36 with hepatic steatosis. The median age was lower in individuals without steatosis (12 years old, IQR: 10.2–14.7) than in those with steatosis (14 years old, IQR: 12–16) (*p* = 0.009, Table S1). The groups of patients stratified according to diagnosis of steatosis were similar with regard to sex (*p* = 0.47, Table S1). The median body mass index-for-age (BMI-for-age) indicator (*p* = 0.012) and waist circumference (*p* = 0.0003) were higher in adolescents with steatosis, but not the frequency of abdominal obesity (*p* = 0.245, Table S1). These adolescents with steatosis also more frequently had hypertension and metabolic syndrome than those from the control group (Table S1).

### 3.2. Changes in Biochemical Parameters in Peripheral Blood Can Distinguish Patients with and without Hepatic Steatosis

**Figure 1:**
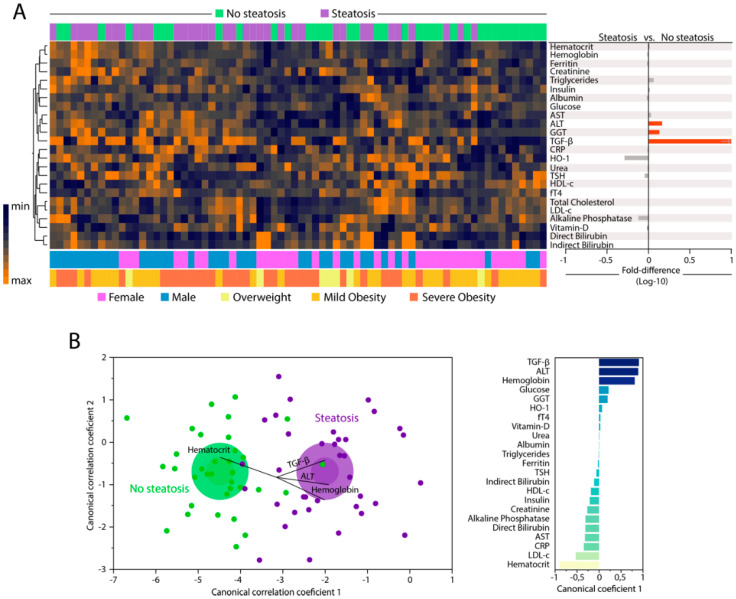
Changes in biochemical proteins of peripheral blood distinguish patients with and without hepatic steatosis. Plasma was assessed in samples from patients without hepatic steatosis (n = 36) and patients with hepatic steatosis (n = 36). Data were Log10 transformed and z-score normalized. (**A**) Left panel: In hierarchical cluster analysis (Ward’s method with 100X bootstrap) was employed to depict the overall expression of plasma proteins in study population. Right panel: Average fold-difference values in plasma proteins levels for patients with hepatic steatosis and without steatosis group. Differences which reached statistical significance with the Mann-Whitney *U* test adjusted for multiple comparisons using the Holm-Bonferroni’s method (Adjusted *p* < 0.05) are represented in colored bars. (**B**) Left panel: In an exploratory approach, a sparse canonical correlation analysis (sCCA) was employed to test whether experimental groups could be distinguished based on correlation profiles of the combined circulating markers. Vector analysis was used to plot the direction of influence of the most significant parameters in the canonical space. Right panel: Canonical coefficient scores were calculated to identify the biomarkers responsible for the difference between groups in the sCCA model. Abbreviations (alphabetic order): ALT: alanine aminotransferase; AST: aspartate aminotransferase; CRP: C-reactive protein; fT4: free thyroxine; GGT: gamma-glutamyl transferase; HDL-c: high density cholesterol; HO-1: heme oxygenase-1; TGF-β: transforming growth factor β; TSH: thyroid-stimulating hormone.

We examined the expression of 25 parameters in peripheral blood to compare the biochemical/inflammatory profiles of individuals with or without hepatic steatosis. A summary of the univariate comparisons is described in Table S2. Unsupervised hierarchical clustering analysis of the log10-transformed and z-score normalized values/concentrations of each parameter revealed no clear distinction of biochemical profiles between the study groups when all markers were examined simultaneously. Individuals with hepatic steatosis exhibited a tendency of decreased levels of HO-1 and alkaline phosphatase, and increased levels of triglycerides, AST, ALT, GGT, and TGF-β (Figure 1A, left panel and Figure S1). Furthermore, fold-difference analysis of the circulating concentrations of each biochemical marker are summarized in Figure 1A, right panel. Among all markers, only median values of ALT, GGT, and TGF-β were statistically distinct between the study groups, with higher values being detected in the group of participants with hepatic steatosis (all with adjusted *p*-values < 0.05). After identifying the consistent changes in the concentrations of biochemical parameters between the clinical groups, we employed a discriminant model using sparse canonical correlation analysis (CCA), as previously described [32,33,34] to test whether the statistical relationships between the biomarkers, rather their concentrations/values themselves, could be used to distinguish the groups. Using this approach, we found that the patients with hepatic steatosis could be distinguished from those without steatosis with high degree of accuracy (area under the curve (AUC) of the receiver operator characteristics (ROC) curve = 0.97 (CI: 0.85–1.0), sensitivity 91% (CI: 77.0–96.7), specificity 86% (72.0–94.0); *p* < 0.0001, Figure 1B, left panel). Assessment of the canonical coefficient values of the CCA model revealed that the most significant markers responsible for the discrimination between individuals with or without steatosis were LDL-c, triglycerides, TGF-β, ALT, HDL-c, and hematocrit (Figure 1B, right panel).

### 3.3. Hepatic Steatosis Leads to Consistent Changes in the Profile of Correlations between Blood Biochemical Parameters

**Figure 2:**
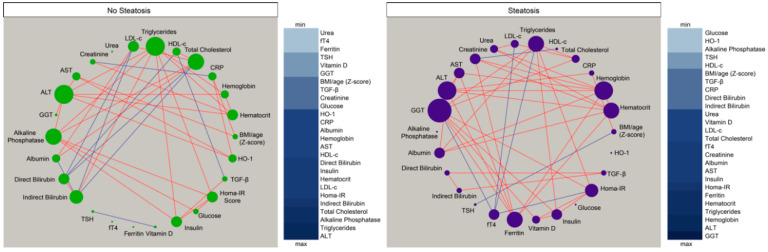
Hepatic steatosis leads consistent changes in correlations between plasma proteins concentrations. Network analysis of the biomarker correlation matrices was performed with bootstrap (100×). Significant correlations (*p* < 0.05) are shown. Each circle represents a different parameter. Circle size infers number of correlations involving each parameter. Lines represent the rho values. Red color infers positive correlation whereas blue color denotes negative correlations. Node analysis heatmap shows the number of statistically significant correlations involving each marker per clinical group. Abbreviations (alphabetic order): ALT: alanine aminotransferase; AST: aspartate aminotransferase; BMI: body mass index; CRP: C-reactive protein; fT4: free thyroxine; GGT: gamma-glutamyl transferase; HDL-c: high density cholesterol; HO-1: heme oxygenase-1; Homa-IR: homeostatic model assessment; TGF-β: transforming growth factor β; TSH: thyroid-stimulating hormone.

Given that we observed in the results presented above that the overall profile of correlations was distinct between the study groups, we then tried to visualize the numerous correlations detected. We employed an approach using network analysis in which Spearman correlations are visualized as connections between the parameters [33,34,35,36,37]. This approach helped us to identify the dynamicity, strength and quality of the relationships between values of the biochemical markers and inflammatory proteins in plasma of the different groups of individuals. We found that networks from the distinct clinical groups displayed differences in complexity and quality of statistical interactions between biochemical markers (Figure 2). Regardless of the clinical group, most of the relevant correlations were positive, meaning that the increases in values of a given marker were followed by heightened concentrations of other biochemical parameters. Importantly, the density of networks (related to number of statistically significant correlations) was higher in the group of patients with hepatic steatosis.

In the group of participants without steatosis, we detected eight negative significant correlations whereas in the group of hepatic steatosis, six negative correlations were observed, highlighting fT4, that exhibited only negative interactions with values of Homa-IR, triglycerides, LDL-c, and GGT. Of note, the markers exhibiting the highest number of significant relationships in the group of individuals without hepatic steatosis were alkaline phosphatase, ALT, and triglycerides (Figure 2, left panel) whereas in the group of patients with steatosis, GGT, ALT, and hemoglobin were the markers with the highest connectivity in the networks (Figure 2, right panel). The analyses also revealed that TGF-β was not the marker with the highest number of correlations in the distinct matrices. However, such marker was related with different parameters in the networks, and with distinct correlation profile (positive vs. negative correlations) (Figure 2), which explained in part why TGF-β was relevant to explain the differences between the study groups described in the discriminant model based on correlations described in Figure 1B. Thus, these results argue that hepatic steatosis is associated with alterations in the profile of correlations between values of the biochemical parameters evaluated here.

### 3.4. Associations between Grade of Hepatic Steatosis and Values of Biochemical Parameters in Peripheral Blood

**Figure 3:**
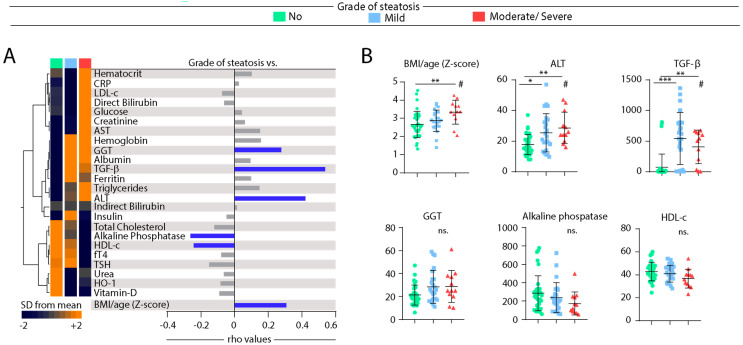
Spearman correlation of biochemical parameters in blood of patients according grade of hepatic steatosis. (**A**) Left panel: Data on each parameter was Log10 transformed. Mean values for each indicated clinical group were z-score normalized and a Hierarchical cluster analysis was performed to illustrate the overall biochemical profiles according the grade of diseases. Right panel: Correlation between grade of hepatic steatosis and biochemical parameters. Spearman correlation analysis was used, and rho values are shown. Blue lines represent correlations with statistical relevance. (**B**) Scatterplots of concentrations of indicated parameter which values presented statistically significant differences between the study groups using the Kruskal-Wallis test with Dunn’s multiple comparisons ad hoc test (* *p* < 0.05, ** *p* < 0.01, *** *p* < 0.001, ns: nonsignificant) # represent statistical significance (*p* < 0.05) of non-parametric linear trend ad hoc test. Bars represent median values whereas whiskers represent the interquartile ranges. Abbreviations (alphabetic order): ALT: alanine aminotransferase; AST: aspartate aminotransferase; BMI: body mass index; CRP: C-reactive protein; fT4: free thyroxine; GGT: gamma-glutamyl transferase; HDL-c: high density cholesterol; HO-1: heme oxygenase-1; TGF-β: transforming growth factor β; TSH: thyroid-stimulating hormone.

We next tested whether gradual increases in the severity of hepatic steatosis are proportional to changes in values of biochemical parameters in blood. Median values of each biochemical parameter per group of individuals stratified according to the grade of hepatic steatosis were calculated (Table S3). These values were log10-transformed, and z-score normalized, to build a heatmap to illustrate trends in data variation (Figure 3A, left panel). This analysis revealed that the individuals stratified based on the grade of steatosis exhibit a distinct expression profile of biochemical parameters. Hierarchical clustering of the biochemical parameters could identify three principal clusters. The first cluster displayed relatively higher values of hematocrit, CRP, LDL-c, direct bilirubin, glucose, creatinine, and AST in those with ultrasonographic diagnosis of moderate or severe steatosis (Figure 3A, left panel). The second cluster exhibited markers that had similar levels between the groups with mild or moderate/severe steatosis, with increased levels of hemoglobin, GGT, and albumin. In addition, TGF-β levels were relatively higher in the group of mild disease, whereas triglycerides and ALT values were higher in moderate/severe steatosis (Figure 3A, left panel). The last cluster showed increased levels of total cholesterol, alkaline phosphatase, HDL-c, fT4, urea, HO-1, and vitamin D in the group of individuals without hepatic steatosis (Figure 3A, left panel).

The concentration values of each biomarker were further examined for direct correlation with the grade of hepatic steatosis using the Spearman correlation test (Figure 3A, right panel). Values of GGT, TGF-β, ALT, and BMI/age presented positive correlation, whereas those of alkaline phosphatase and HDL-c were negatively correlated, with grade of hepatic steatosis (Figure 3A, right panel). Figure 3B shows scatter plots with distribution of the six parameters which were statistically associated with hepatic steatosis severity in the correlation analysis. We observed that median values of BMI, ALT, and TGF-β were increased in individuals with steatosis using the Kruskal-Wallis test. Median values of GGT, alkaline phosphatase and HDL-c did not exhibit a clear variation following the degree of steatosis. Of note, although the concentrations of TGF-β were higher in individuals with HS, the values were indistinguishable between patients with mild disease and those with moderate/severe presentation (Figure 3B). Furthermore, we examined in more detail the correlations between values of BMI, ALT, and TGF-β when all the study participants were investigated as a single group, regardless of steatosis (Figure S2). Importantly, concentration of both TGF-β and ALT were positively correlated with BMI/age Z-score values (*r* = 0.23; *p* = 0.04 and *r* = 0.34; *p* = 0.003, respectively). Similar analysis also revealed a positive correlation between levels of TGF-β and ALT (*r* = 0.31; *p* = 0.0006).

### 3.5. Patients with Hepatic Steatosis Display Higher Degree of Biochemical Perturbation in Peripheral Blood

**Figure 4:**
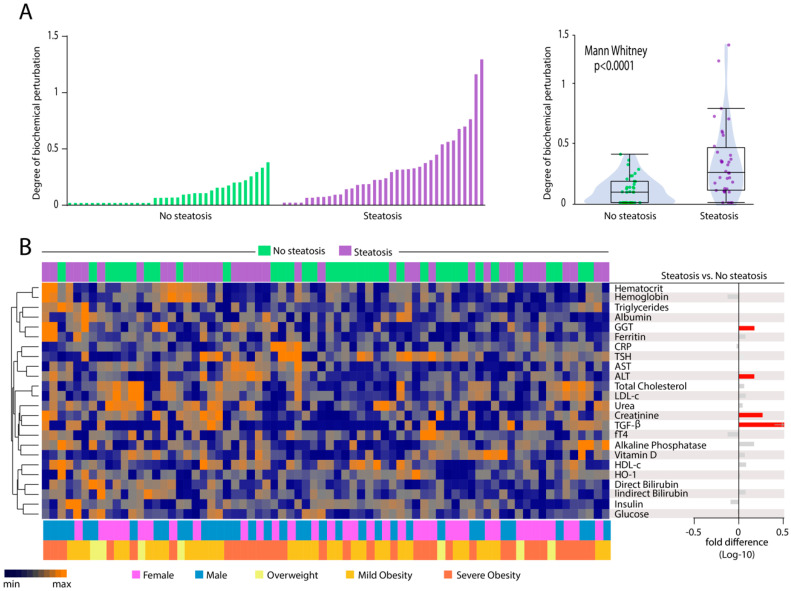
Hepatic steatosis is associated with increases in degree of biochemical perturbation. (**A**) Left panel: Histograms show the single sample degree of biochemical perturbation (DBP) score values relative to each study group as indicated. Right panel: Box plots represent the distribution of the DBP between study groups. Values were compared between patients with and without steatosis using Mann Whitney *U* test. (**B**) Left panel: A hierarchical cluster analysis (Ward’s method) was employed to show the molecular degree of perturbation of each biochemical marker. Right panel: Average fold-difference values in DBP for patients with hepatic steatosis and without steatosis group. Abbreviations (alphabetic order): ALT: alanine aminotransferase; AST: aspartate aminotransferase; CRP: C-reactive protein; fT4: free thyroxine; GGT: gamma-glutamyl transferase; HDL-c: high density cholesterol; HO-1: heme oxygenase-1; TGF-β: transforming growth factor β; TSH: thyroid-stimulating hormone.

To deepen the analyses in overall disturbances in biochemical profiles, we calculated the Degree of Biochemical Perturbation (DBP, as described in Methods) (Figure 4A), which is an adaptation from the molecular degree of perturbation previously published [33,34,35]. We found that patients with hepatic steatosis exhibited substantial increase in DBP score values compared to those without steatosis (*p* < 0.0001). We next employed a hierarchical clustering analysis using the DBP values calculated for each biomarker (Figure 4B). Although the overall DBP values were on average higher in individuals with hepatic steatosis, the individual DBP values calculated for each biomarker could not clearly distinct the subgroups of participants stratified by occurrence of steatosis (Figure 4B), degree of steatosis (Figure S3) or degree of obesity (Figure S4) as well as by sex (Figure 4B). Only perturbations of ALT, GGT, creatinine, and TGF-β were statistically higher in steatosis (Figure 4B, right panel). Our findings reinforce the hypothesis that hepatic steatosis is hallmarked by an altered overall biochemical profile in peripheral blood.

### 3.6. Heightened Plasma Levels of TGF-β Hallmarks Hepatic Steatosis

**Figure 5:**
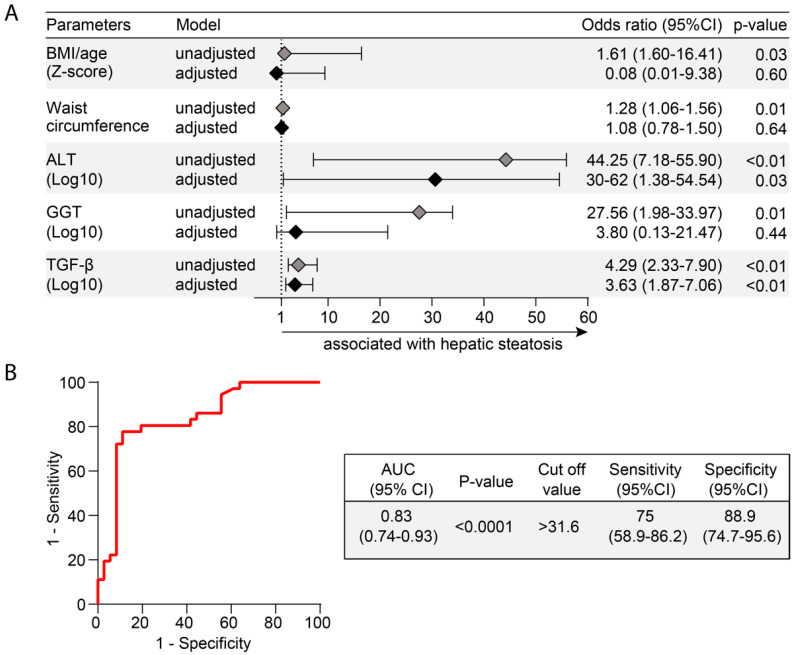
Biochemical parameters associated with hepatic steatosis. (**A**) Multivariable regression model of variables that were statistically significant (*p* < 0.05) in univariate comparisons (see univariate comparisons in Tables S1 and S2). (**B**) Receiver Operator Characteristic (ROC) curves were employed to test the performance of TGF-β to distinguish patients with or without hepatic steatosis. Abbreviations (alphabetic order): ALT: alanine aminotransferase; AST: aspartate aminotransferase; AUC: area under the curve; BMI: body mass index; 95% CI: 95% confidence interval; GGT: gamma-glutamyl transferase; TGF-β: transforming growth factor β.

Finally, we employed a multivariate analysis to identify biochemical parameters that could be independently associated with hepatic steatosis. Markers that displayed *p*-value ≤ 0.2 in univariate comparisons between the groups of participants with or without hepatic steatosis were included in the multivariate model (Tables S1 and S2 and Figure 5A). Importantly, after adjustment for sex, age, BMI/age z score, and waist circumference, increases in 1-log in TGF-β plasma concentrations were associated with 3.63 higher odds of hepatic steatosis. We used a ROC curve analysis to calculate accuracy of this potential biomarker of hepatic steatosis. We found that TGF-β plasma concentrations (cut off value > 31.6 pg/mL) had a total accuracy of 83% (area under the curve (AUC) of the ROC (95% CI) = 0.83 (0.74–0.93), with 75% sensitivity and 88.9% specificity to diagnose hepatic steatosis.

## 4. Discussion

NASH has recently become the first cause of cirrhosis and liver-related deaths worldwide [38] and remains an important public health problem. The prevalence of NASH is increasing along with the rate of overweight and obesity with the metabolic syndrome [5,8,17]. The detailed description of biochemical profiles of NASH may contribute to understanding of pathophysiological processes and could help the clinical management of these patients. In the present study, we show that obese adolescents with hepatic steatosis exhibit a distinct biochemical profile in peripheral blood. Interestingly, our analyses reveal that these changes in the expression profile of such biochemical parameters are able to distinguish steatosis patients from those without this condition. Additionally, to the best of our knowledge, this study represents the first attempt to establish an association between elevated levels on TGF-β and hepatic steatosis in obese adolescents. Furthermore, the results indicate an important biochemical imbalance in steatosis patients, which could guide further studies of new diagnostics tools and therapeutic targets in order to optimize clinical management.

Inflammation is a coordinated process that occurs with synchronized changes in production of several immunologic and non-immunologic markers [6,39]. Although studies have shown the activation of the inflammatory response in hepatocytes after the lipid accumulation in liver is the principal trigger to NASH [40], the potential influence of other biochemical markers in clinical outcomes is still unknown. In steatosis, activation on the inflammatory pathways is observed in early stage of diseases [41,42] with release of known markers due to their association with injury in hepatic tissue. As expected, our results revealed substantially increases in levels of ALT, GGT, and TGF-β in patients with steatosis compared to those without steatosis, and all of such makers have been associated with NASH prediction [43]. The consistent changes in expression of biochemical parameters observed in steatosis group are able to distinguish the different clinical groups compared in the present study, detected by a discriminant analysis based on canonical correlations. Of note, canonical coefficient analysis identified candidate biomarkers likely responsible for the distinction between no steatosis and steatosis groups, such as TGF-β, ALT, hemoglobin, and hematocrit.

Importantly, in a previous report with adult patients presenting hepatic steatosis caused by obesity or alcohol, TGF-β has been strongly associated with this clinical outcome [44]. Our findings suggest that the disturbed biochemical profile detected in steatosis patients was so substantially different that could reliably distinguish the different study groups. It is possible that the top markers describe above, mainly TGF-β, play a significant role in the pathogenesis of liver injury induced during NASH. TGF-β is secreted in response to cell damage, and plays the dominant role in the mediation of fibrosis, through its contribution to the activation of stellate cells and their production of extracellular matrix proteins. As detailed recently, TGF-β desirable acts through activin receptor-like kinase 5 (ALK5) type I receptor (TGF-β RI) and the TGF-β type II receptor (TGF-β RII) [45]. The serum level of TGF-β and the tissue level of TGF-β receptor mRNA can be measured and used as diagnostic and prognostic markers for liver diseases [25,45]. This growth factor has many actions in the liver, including: (i) fibrogenesis (ii) growth inhibition (of normal hepatocytes and stellate cells) (iii) mitogenesis (iv) pro-apoptosis and (v) chemo-attraction. Described as a biochemical marker for progression to NASH, there is no description of studies involving the marker in the pediatric age group [26,46]. Considering the crucial role in the development and progression of liver fibrosis [45], we could hypothesize that the majority of obese adolescents studied here present or will evolve to NASH.

Here, two hypotheses should be considered to explain the meaning of increased TGF-β and if is it really associated to steatosis alone or in fact is a predictor for fibrosis and NASH. The extremely significant elevation of TGF-β levels among our steatosis participants can represent a spectrum of patients who already have steatohepatitis and could not be diagnosed by the impossibility of performing liver biopsy. In fact, we observed that one third of participants with hepatic steatosis (n = 12/36) had ALT ≥ 30 U/L (data not shown) [47]. Moreover, we observed a significant correlation between ALT and TGF-β levels. This hypothesis has some evidence in literature among adults [46]. However, we could not find previously reported data on pediatric patients that could mirror the findings presented here. Further investigation with sequential monitoring of ALT levels and other biochemical parameters and imaging markers and/or a liver biopsy could help in clarifying this possibility in the future. On the other hand, elevated levels of TGF-β may also represent patients only with hepatic steatosis without NASH. In fact, 20 of 36 patients exhibited ALT levels ≤ 25 U/L [1]. Although ALT is not a definitive biochemical marker to NASH, it is commonly used as indicator of hepatic damage in clinical medicine [47]. Thus, lower levels can be a proxy of absence/minimal hepatic tissue damage. Furthermore, a faster and more severe evolution/progression of the disease is observed in pediatric NAFLD patients compared to adults [11,48,49] and we hypothesize that the elevated levels of TGF-β could be a marker of this condition in pediatric population, although we failed to formally demonstrate that levels of this biomarker directly reads progression of hepatis steatosis. A follow-up of these children and adolescents is underway to clarify this question.

An important contribution of our study was the assessment of the quality and magnitude of the statistical interactions between the biochemical parameters. Spearman correlation matrices have been used in several diseases marked by both acute and chronic inflammation [33,36,50,51] and suggest that changes in the correlation network profile indicate alteration of systemic inflammation status. Indeed, our correlation analyses revealed different profiles between the clinical groups with intense interactions being detected in the group of hepatic steatosis. Interesting, this group was marked by the presence of two positive correlation involving important markers associated with liver diseases. We hypothesize that hepatic steatosis is characterized by a hepatic inflammatory milieu that leads to consistent changes in systemic concentrations of biochemical parameters.

Next, we evaluated the correlation between grade of hepatic steatosis and the concentrations/values of biochemical parameters as well as of BMI. We found that linear increases occurred in BMI and ALT according steatosis severity. Although the levels of TGF-β presented higher expression values among children and adolescents with steatosis, these concentrations were not different between patients with mild and those with moderate/severe disease. This observation reinforces the idea that TGF-β may be more useful to identify presence steatosis than to infer prognosis/disease progression. In addition, we found that decreases in alkaline phosphatase and HDL-c values were proportional to gradual augmentation of steatosis severity. Further studies are warranted to delineate the molecular mechanisms underlying these associations.

Another important contribution of our results is the assessment to the degree of biochemical perturbation. To our knowledge, no previous study has estimated the global biochemical disturbance in hepatic steatosis patients. We demonstrated that hepatic steatosis is indeed linked to overall higher biochemical imbalance, which was shown to be even more significant in the individuals with obesity, both mild or severe. Our result reinforces the role of obesity, associated with the consequent degree of insulin resistance as a potential factor driving chronic inflammation and implicated in homeostatic disturbance underlying steatosis [52,53].

Finally, using multivariable analysis, we examined the biochemical parameters most associated with hepatic steatosis. Among such parameters, the TGF-β was the most robust in the predictive model employed. Importantly, to minimize the possible effects of extra-hepatic TGF-β production, as adipose tissue [54], and because levels of this cytokine were positively correlated with values of BMI in the study population, our model was adjusted for BMI/age Z-score and waist circumference. After the adjustment, increases of 1 Log of TGF-β levels remained independently associated with 3.63 increased risk of steatosis development. Within the biochemical parameters used in this multivariable analysis was ALT. Spearman correlation analysis revealed a positive association between concentrations of ALT and those of TGF-β in the entire study population, reinforcing our hypotheses that the higher levels of TGF-β found here are a consequence of liver injury, and not by other tissues production. Of note, it has been demonstrated the importance of TGF-β as a critical factor determining steatohepatitis [26,46,55]. Taken together, these data suggest that in patients with hepatic steatosis, there is an up-regulation of markers involved in persistent inflammation, which may contribute to the pathophysiology of hepatic steatosis. Additional studies are needed to investigate the immune activation profile of these patients, in order to suggest new therapeutic targets. In that sense, our finding on the potential use of TGF-β plasma levels as an accurate biomarker of hepatic steatosis seems promising. Furthermore, this finding is especially important given that the adolescents studied here were mostly severely obese and traditional biochemical markers such as triglycerides, HDL-c, insulinemia, and HOMA-IR were not able to adequately identify presence of hepatic steatosis. Usually, the assessment of the disease severity is based on noninvasive radiologic examination, specifically ultrasonography [56]. However, this method is subject to errors depending on the operator. In this context, TGF-β could emerge as a potential tool in the diagnosis of steatosis, maybe associated with ultrasonography, but without a clear relevant impact in the evaluation of the grade of hepatic steatosis. Future studies are needed to test and validate the accuracy of TGF-β, alone or in association with other non-invasive methods, in a larger number of patients.

Our study has some limitations. The limited sample size, the absence of measurements of other inflammatory cytokines to establish a more detailed profile of the metabolic disorder, and the absence of a histological method for the diagnosis of NASH for a comparison with the results of the non-invasive evaluation performed. In addition, our study design was cross-sectional. Thus, further longitudinal studies are therefore needed in order to assess the chronological relationship between TGF-β levels and occurrence of NAFLD to clarify the probable impact of reverse causality. Independent of the direction of this association, further attention should be paid to the good accuracy observed between elevated levels of TGF-β and the presence of hepatic steatosis in children and adolescents.

## 5. Conclusions

Our findings reveal that hepatic steatosis patients exhibit a distinct biosignature of biochemical parameters, and that TGF-β is an important biomarker that could help evaluate individuals with hepatic steatosis, in association with other non-invasive techniques such as ultrasound.

## Supplementary Materials

The following are available online at https://www.mdpi.com/2072-6643/12/6/1650/s1. Table S1: Characteristics of the study participants. Table S2: Median values of measured plasma markers. Table S3: Concentration of parameters according to grade of hepatic steatosis. Figure S1: Overall profile of concentrations of biochemical parameters according to occurrence of hepatic steatosis and degree of obesity. Figure S2: Correlations between values of BMI/age, ALT and TGF-β in the study population. Figure S3: Degree of biochemical perturbation of each biomarker according to grade of hepatic steatosis. Figure S4: Degree of biochemical perturbation of each biomarker according to degree of obesity.

## References

[1] Vos M.B., Abrams S.H., Barlow S.E., Caprio S., Daniels S.R., Kohli R., Mouzaki M., Sathya P., Schwimmer J.B., Sundaram S.S. (2017). NASPGHAN Clinical Practice Guideline for the Diagnosis and Treatment of Nonalcoholic Fatty Liver Disease in Children: Recommendations from the Expert Committee on NAFLD (ECON) and the North American Society of Pediatric Gastroenterology, Hepatology and Nutrition (NASPGHAN). J. Pediatr. Gastroenterol. Nutr..

[2] Sheka A.C., Adeyi O., Thompson J., Hameed B., Crawford P.A., Ikramuddin S. (2020). Nonalcoholic Steatohepatitis: A Review. JAMA.

[3] Nobili V., Alkhouri N., Alisi A., Della Corte C., Fitzpatrick E., Raponi M., Dhawan A. (2015). Nonalcoholic fatty liver disease: A challenge for pediatricians. JAMA Pediatr..

[4] Anderson E.L., Howe L.D., Jones H.E., Higgins J.P., Lawlor D.A., Fraser A. (2015). The Prevalence of Non-Alcoholic Fatty Liver Disease in Children and Adolescents: A Systematic Review and Meta-Analysis. PLoS ONE.

[5] Draijer L., Benninga M., Koot B. (2019). Pediatric NAFLD: An overview and recent developments in diagnostics and treatment. Expert Rev. Gastroenterol. Hepatol..

[6] Mann J.P., Valenti L., Scorletti E., Byrne C.D., Nobili V. (2018). Nonalcoholic Fatty Liver Disease in Children. Semin. Liver Dis..

[7] Assuncao S.N.F., Sorte N.C.B., Alves C.D., Mendes P.S.A., Alves C.R.B., Silva L.R. (2017). Nonalcoholic fatty liver disease (NAFLD) pathophysiology in obese children and adolescents: Update. Nutr. Hosp..

[8] Nobili V., Alisi A., Valenti L., Miele L., Feldstein A.E., Alkhouri N. (2019). NAFLD in children: New genes, new diagnostic modalities and new drugs. Nat. Rev. Gastroenterol. Hepatol..

[9] Clemente M.G., Mandato C., Poeta M., Vajro P. (2016). Pediatric non-alcoholic fatty liver disease: Recent solutions, unresolved issues, and future research directions. World J. Gastroenterol..

[10] Fang Y.L., Chen H., Wang C.L., Liang L. (2018). Pathogenesis of non-alcoholic fatty liver disease in children and adolescence: From “two hit theory” to “multiple hit model”. World J. Gastroenterol..

[11] Holterman A.X., Guzman G., Fantuzzi G., Wang H., Aigner K., Browne A., Holterman M. (2013). Nonalcoholic fatty liver disease in severely obese adolescent and adult patients. Obesity (Silver Spring).

[12] Holterman A., Gurria J., Tanpure S., DiSomma N. (2014). Nonalcoholic fatty liver disease and bariatric surgery in adolescents. Semin. Pediatr. Surg..

[13] Rafiq N., Bai C., Fang Y., Srishord M., McCullough A., Gramlich T., Younossi Z.M. (2009). Long-term follow-up of patients with nonalcoholic fatty liver. Clin. Gastroenterol. Hepatol..

[14] Vajro P., Lenta S., Socha P., Dhawan A., McKiernan P., Baumann U., Durmaz O., Lacaille F., McLin V., Nobili V. (2012). Diagnosis of nonalcoholic fatty liver disease in children and adolescents: Position paper of the ESPGHAN Hepatology Committee. J. Pediatr. Gastroenterol. Nutr..

[15] Uppal V., Mansoor S., Furuya K.N. (2016). Pediatric Non-alcoholic Fatty Liver Disease. Curr. Gastroenterol. Rep..

[16] Day C.P., James O.F. (1998). Hepatic steatosis: Innocent bystander or guilty party?. Hepatology.

[17] Esler W.P., Bence K.K. (2019). Metabolic Targets in Nonalcoholic Fatty Liver Disease. Cell Mol. Gastroenterol. Hepatol..

[18] Cohen J.C., Horton J.D., Hobbs H.H. (2011). Human fatty liver disease: Old questions and new insights. Science.

[19] Machado M.V., Diehl A.M. (2016). Pathogenesis of Nonalcoholic Steatohepatitis. Gastroenterology.

[20] Thamer C., Tschritter O., Haap M., Shirkavand F., Machann J., Fritsche A., Schick F., Haring H., Stumvoll M. (2005). Elevated serum GGT concentrations predict reduced insulin sensitivity and increased intrahepatic lipids. Horm. Metab. Res..

[21] Assunção S.N.F., Sorte N.C.A.B., Alves C.A.D., Mendes P.S.A., Alves C.R.B., Silva L.R. (2018). Inflammatory cytokines and non-alcoholic fatty liver disease (NAFLD) in obese children and adolescents. Nutr. Hosp..

[22] Drummond G.S., Baum J., Greenberg M., Lewis D., Abraham N.G. (2019). HO-1 overexpression and underexpression: Clinical implications. Arch. Biochem. Biophys..

[23] Yuan X.W., Li D.D., Liu L.D., Zhang Y., Zhao W., Cui L.Y., Yang Y., Nan Y.M. (2019). Application of heme oxygenase 1 in the diagnosis of non-alcoholic fatty liver disease. Zhonghua Gan Zang Bing Za Zhi.

[24] Abraham N.G., Junge J.M., Drummond G.S. (2016). Translational Significance of Heme Oxygenase in Obesity and Metabolic Syndrome. Trends Pharm. Sci..

[25] Gressner A.M., Weiskirchen R., Breitkopf K., Dooley S. (2002). Roles of TGF-beta in hepatic fibrosis. Front. Biosci..

[26] Flisiak R., Pytel-Krolczuk B., Prokopowicz D. (2000). Circulating transforming growth factor beta(1) as an indicator of hepatic function impairment in liver cirrhosis. Cytokine.

[27] de Onis M., Garza C., Onyango A.W., Rolland-Cachera M.F., de pédiatrie l.C.d.d.f. (2009). WHO growth standards for infants and young children. Arch. Pediatr..

[28] Hamaguchi M., Kojima T., Itoh Y., Harano Y., Fujii K., Nakajima T., Kato T., Takeda N., Okuda J., Ida K. (2007). The severity of ultrasonographic findings in nonalcoholic fatty liver disease reflects the metabolic syndrome and visceral fat accumulation. Am. J. Gastroenterol..

[29] Freedman D.S., Serdula M.K., Srinivasan S.R., Berenson G.S. (1999). Relation of circumferences and skinfold thicknesses to lipid and insulin concentrations in children and adolescents: The Bogalusa Heart Study. Am. J. Clin. Nutr..

[30] Flynn J.T., Kaelber D.C., Baker-Smith C.M., Blowey D., Carroll A.E., Daniels S.R., de Ferranti S.D., Dionne J.M., Falkner B., Flinn S.K. (2017). Clinical Practice Guideline for Screening and Management of High Blood Pressure in Children and Adolescents. Pediatrics.

[31] Zimmet P., Alberti G., Kaufman F., Tajima N., Silink M., Arslanian S., Wong G., Bennett P., Shaw J., Caprio S. (2007). The metabolic syndrome in children and adolescents. Lancet.

[32] Mayer-Barber K.D., Andrade B.B., Oland S.D., Amaral E.P., Barber D.L., Gonzales J., Derrick S.C., Shi R., Kumar N.P., Wei W. (2014). Host-directed therapy of tuberculosis based on interleukin-1 and type I interferon crosstalk. Nature.

[33] Vinhaes C.L., Arriaga M.B., de Almeida B.L., Oliveira J.V., Santos C.S., Calcagno J.I., Carvalho T.X., Giovanetti M., Alcantara L.C.J., de Siqueira I.C. (2020). Newborns with Zika virus-associated microcephaly exhibit marked systemic inflammatory imbalance. J. Infect. Dis..

[34] Vinhaes C.L., Oliveira-de-Souza D., Silveira-Mattos P.S., Nogueira B., Shi R., Wei W., Yuan X., Zhang G., Cai Y., Barry C.E. (2019). Changes in inflammatory protein and lipid mediator profiles persist after antitubercular treatment of pulmonary and extrapulmonary tuberculosis: A prospective cohort study. Cytokine.

[35] Oliveira-de-Souza D., Vinhaes C.L., Arriaga M.B., Kumar N.P., Cubillos-Angulo J.M., Shi R., Wei W., Yuan X., Zhang G., Cai Y. (2019). Molecular degree of perturbation of plasma inflammatory markers associated with tuberculosis reveals distinct disease profiles between Indian and Chinese populations. Sci. Rep..

[36] Cruz L.A.B., Moraes M.O.A., Queiroga-Barros M.R., Fukutani K.F., Barral-Netto M., Andrade B.B. (2019). Chronic hepatitis B virus infection drives changes in systemic immune activation profile in patients coinfected with Plasmodium vivax malaria. PLoS Negl. Trop. Dis..

[37] Andrade B.B., Singh A., Narendran G., Schechter M.E., Nayak K., Subramanian S., Anbalagan S., Jensen S.M., Porter B.O., Antonelli L.R. (2014). Mycobacterial antigen driven activation of CD14++CD16- monocytes is a predictor of tuberculosis-associated immune reconstitution inflammatory syndrome. PLoS Pathog..

[38] Suzuki A., Diehl A.M. (2017). Nonalcoholic Steatohepatitis. Annu. Rev. Med..

[39] Ore A., Akinloye O.A. (2019). Oxidative Stress and Antioxidant Biomarkers in Clinical and Experimental Models of Non-Alcoholic Fatty Liver Disease. Medicina (Kaunas).

[40] Angulo P. (2002). Nonalcoholic fatty liver disease. N. Engl. J. Med..

[41] Brenner C., Galluzzi L., Kepp O., Kroemer G. (2013). Decoding cell death signals in liver inflammation. J. Hepatol..

[42] Hirsova P., Ibrahim S.H., Krishnan A., Verma V.K., Bronk S.F., Werneburg N.W., Charlton M.R., Shah V.H., Malhi H., Gores G.J. (2016). Lipid-Induced Signaling Causes Release of Inflammatory Extracellular Vesicles from Hepatocytes. Gastroenterology.

[43] Bedogni G., Bellentani S., Miglioli L., Masutti F., Passalacqua M., Castiglione A., Tiribelli C. (2006). The Fatty Liver Index: A simple and accurate predictor of hepatic steatosis in the general population. BMC Gastroenterol..

[44] Chiappini F., Barrier A., Saffroy R., Domart M.C., Dagues N., Azoulay D., Sebagh M., Franc B., Chevalier S., Debuire B. (2006). Exploration of global gene expression in human liver steatosis by high-density oligonucleotide microarray. Lab. Investig..

[45] Nair B., Nath L.R. (2020). Inevitable role of TGF-β1 in progression of nonalcoholic fatty liver disease. J. Recept. Signal Transduct..

[46] Khalid M., Hadhoud E., Ayman A.-R., Fawzy E., Fawzy M. (2014). Transforming Growth Factor Beta One and Non Alcoholic Fatty Liver Disease. Afro-Egypt J. Infect. Endem. Dis..

[47] Hadizadeh F., Faghihimani E., Adibi P. (2017). Nonalcoholic fatty liver disease: Diagnostic biomarkers. World J. Gastrointest. Pathophysiol..

[48] Lavine J.E., Schwimmer J.B., Van Natta M.L., Molleston J.P., Murray K.F., Rosenthal P., Abrams S.H., Scheimann A.O., Sanyal A.J., Chalasani N. (2011). Effect of vitamin E or metformin for treatment of nonalcoholic fatty liver disease in children and adolescents: The TONIC randomized controlled trial. JAMA.

[49] McPherson S., Hardy T., Henderson E., Burt A.D., Day C.P., Anstee Q.M. (2015). Evidence of NAFLD progression from steatosis to fibrosing-steatohepatitis using paired biopsies: Implications for prognosis and clinical management. J. Hepatol..

[50] Manion M., Andrade B.B., DerSimonian R., Gu W., Rupert A., Musselwhite L.W., Sierra-Madero J.G., Belaunzaran-Zamudio P.F., Sanne I., Lederman M.M. (2017). Country of residence is associated with distinct inflammatory biomarker signatures in HIV-infected patients. J. Virus Erad..

[51] Mendonca V.R., Queiroz A.T., Lopes F.M., Andrade B.B., Barral-Netto M. (2013). Networking the host immune response in Plasmodium vivax malaria. Malar J..

[52] Wong R.J., Tran T., Kaufman H., Niles J., Gish R. (2019). Increasing metabolic co-morbidities are associated with higher risk of advanced fibrosis in nonalcoholic steatohepatitis. PLoS ONE.

[53] Kojima S., Watanabe N., Numata M., Ogawa T., Matsuzaki S. (2003). Increase in the prevalence of fatty liver in Japan over the past 12 years: Analysis of clinical background. J. Gastroenterol..

[54] Lee M.J. (2018). Transforming growth factor beta superfamily regulation of adipose tissue biology in obesity. Biochim. Biophys. Acta Mol. Basis Dis..

[55] Alisi A., Nobili V., Ceccarelli S., Panera N., De Stefanis C., De Vito R., Vitali R., Bedogni G., Balsano C., Cucchiara S. (2014). Plasma high mobility group box 1 protein reflects fibrosis in pediatric nonalcoholic fatty liver disease. Expert Rev. Mol. Diagn..

[56] Lee Y.S., Yoo Y.J., Jung Y.K., Kim J.H., Seo Y.S., Yim H.J., Kim I.H., Lee S.Y., Kim B.H., Kim J.W. (2020). Multiparametric MR is a Valuable Modality for Evaluating Disease Severity of Nonalcoholic Fatty Liver Disease. Clin. Transl. Gastroenterol..

