# Supplementary material for: Heightened Plasma Levels of Transforming Growth Factor Beta (TGF-β) and Increased Degree of Systemic Biochemical Perturbation Characterizes Hepatic Steatosis in Overweight Pediatric Patients: A Cross-Sectional Study
Source: Nutrients. 2020 Jun 2;12(6):1650. doi: 10.3390/nu12061650 (PMC7352859; doi:10.3390/nu12061650)
Supplement: Supplementary file 1 [file nutrients-12-01650-s001.pdf]

Table S1. Characteristics of the study participants

Characteristic	No steatosis	Steatosis	*P*-value
N	36	36	
Age – y	12.5 (10.2-14.7)	14.0 (12.0-16.0)	**0.0096**
Male – no. (%)	16 (44.4)	20 (55.7)	0.4798
Metabolic Syndrome – no. (%)	2 (5.6)	8 (22.2)	0.0850
Severe obesity – no. (%)	12 (33.3)	19 (52.8)	0.1530
BMI/age- (Z- score)	2.59 (2.17 – 3.14)	3.01 (2.57 – 3.44)	**0.0122**
WC > 90th percentile – no. (%)	26 (72.2)	31 (86.1)	0.2450
Waist circumference (WC) - cm	87.8 (82.3 – 97.5)	101.0 (92.5 – 110.1)	**0.0003**
Systemic Arterial Hypertension - no. (%)	2 (5.6)	10 (27.8)	**0.0238**
Systolic blood pressure - mmHg	115.5 (107.5 – 121.5)	114.5 (105.5 – 123.0)	0.8791
Diastolic blood pressure - mmHg	70.0 (64.5 – 73.5)	70.0 (62.5 – 79.5)	0.5614

Data represent median and interquartile range (continuous variables) or frequency (categorical variables). The Mann-Whitney *U* test was used to compare age, anthropometric data and blood pressure distribution between the groups and the Fisher's exact test was used to compare frequency of male individuals and comorbidities frequency between the groups. *P*-values in bold font are statistically significant.

Table S2. Median values of measured plasma markers

Biomarker	Unit	No steatosis	Steatosis	*P*-value
Hematocrit	%	38.9 (36.5-40.4)	38.8 (37.5-41.5)	0.4014
Hemoglobin	g/dL	13.1 (12.3-13.8)	13.2 (12.7-14.2)	0.1969
Glucose	mg/dL	87.3 (84.1-91.8)	87.3 (84.4-91.5)	0.9282
Insulin	UI/mL	15.2 (9.4-24.0)	16.1 (9.5-22.4)	0.7270
Homa-IR	-	3.4 (2.2 – 4.9)	3.5 (2.1 – 4.5)	0.6769
Total Cholesterol	mg/dL	159.0 (140.5-180.0)	157.5 (123.5-181.5)	0.2414
HDL-c	mg/dL	41.0 (37.2-47.0)	39.35 (33.0-45.7)	0.0953
LDL-c	mg/dL	89.6 (79.8-113.5)	91.5 (71.0-108.3)	0.3440
Triglycerides	mg/dL	82.5 (71.0-124.3)	97.5 (79.0-145.5)	0.1990
Urea	mg/dL	22.0 (19.0-26.7)	21.0 (18.0-25.5)	0.4428
Creatinine	mg/dL	0.63 (0.60-0.70)	0.64 (0.57-0.79)	0.6563
AST	U/L	19.1 (17.0-23.9)	21.0 (18.0-26.5)	0.3489
ALT	U/L	16.0 (12.2-21.8)	24.4 (18.4-33.6)	**0.0008**
GGT	U/L	18.0 (16.0-23.0)	25.0 (17.2-36.5)	**0.0185**
Alkaline Phosphatase	U/L	256.2 (134.3-327.5)	189.5 (89.5-264.8)	0.0514
Albumin	g/dL	4.4 (4.2-4.6)	4.45 (4.3-4.7)	0.3540
Direct Bilirubin	g/dL	0.16 (0.13-0.22)	0.16 (0.12-0.20)	0.4770
Indirect Bilirubin	g/dL	0.17 (0.10-0.24)	0.17 (0.10-0.29)	0.9955
TSH	µUL/ml	2.2 (1.6-3.0)	2.0 (1.45-3.23)	0.5246
fT4	ng/dL	1.2 (1.0-1.3)	1.16 (1.06-1.26)	0.5580
Ferritin	ng/mL	68.1 (43.7-81.9)	72.2 (42.4-108.5)	0.2771
Vitamin D	ng/mL	21.7 (19.1-24.8)	20.5 (18.1-24.3)	0.4850
us-CRP	mg/dL	0.25 (0.10-0.62)	0.25 (0.09-0.50)	0.9148
TGF-β	pg/mL	4.58 (0.11-15.3)	539.0 (31.3-706.1)	**0.0001**
HO-1	pg/mL	47.9 (12.1-98.4)	24.1 (11.1-68.9)	0.3384

The Mann-Whitney *U* test was used to compare continuous variables between the groups. P-values in bold font were statistically significant.

Table S3. Concentration of parameters according to grade of hepatic steatosis

Parameter	Unit	Hepatic steatosis			*P*-value	Post-test result
		No	Mild	Moderate/ Severe		
Body Mass Index	Kg/m^2	28.6 (26.2-33.2)	31.4 (28.6-34.9)	38.1 (35.1-40.1)	**0.0005**	**b**
BMI/age	Z score	2.6 (2.2 – 3.1)	2.9 (2.6 – 3.3)	3.4 (3.1 – 3.7)	**0.0080**	**b**
Hematocrit	%	38.9 (36.5-40.4)	38.4 (37.4-41.9)	39.4 (37.8-40.9)	0.6665	ns
Hemoglobin	g/dL	13.1 (12.3-13.8)	13.2 (12.6-14.4)	13.2 (12.8-14.2)	0.3985	ns
Glucose	mg/dL	87.3 (84.1-91.8)	85.9 (83.8-90.5)	89.7 (86.5-91.8)	0.2948	ns
Insulin	UI/mL	15.2 (9.4-24.0)	16.8 (9.34-22.7)	15.4 (9.5-21.4)	0.8844	ns
Homa-IR	-	3.4 (2.2 – 4.9)	3.7 (2.1 – 4.8)	3.3 (2.6 – 4.5)	0.8574	ns
Total Cholesterol	mg/dL	159.0 (140.5-180.0)	157.5 (119.3-181.5)	154 (135.8-180.5)	0.4939	ns
HDL	mg/dL	41.0 (37.2-47.0)	40.5 (34.7-46.8)	37.5 (30.5-40.0)	0.0736	ns
LDL	mg/dL	89.6 (79.8-113.5)	86.5 (68.0-108.3)	95.5 (83.3-116.0)	0.4842	ns
Triglycerides	mg/dL	82.5 (71.0-124.3)	97.5 (74.5-195.8)	107.5 (81.0-134.3)	0.4278	ns
Urea	mg/dL	22.0 (19.0-26.7)	21.0 (17.0-27.7)	21.5 (19.0-25.5)	0.6584	ns
Creatinine	mg/dL	0.63 (0.60-0.70)	0.63 (0.57-0.78)	0.69 (0.55-0.80)	0.8367	ns
AST	U/L	19.1 (17.0-23.9)	19.5 (17.0-27.7)	22.5 (20.2-24.7)	0.2614	ns
ALT	U/L	16.0 (12.2-21.8)	22.0 (15.0-33.6)	25.0 (21.0-37.5)	**0.0016**	**a; b**
GGT	U/L	18.0 (16.0-23.0)	25.0 (17.0-35.0)	25.0 (20.5-37.7)	0.0576	ns
Alkaline Phosphatase	U/L	256.2 (134.3-327.5)	206.4 (104.0-310.8)	138.0 (81.0-248.5)	0.0662	ns
Albumin	g/dL	4.4 (4.2-4.6)	4.45 (4.30-4.70)	4.45 (4.15-4.60)	0.6382	ns
Direct Bilirubin	g/dL	0.16 (0.13-0.22)	0.15 (0.11-0.19)	0.17 (0.12-0.21)	0.7231	ns
Indirect Bilirubin	g/dL	0.17 (0.10-0.24)	0.17 (0.05-0.25)	0.17 (0.10-0.42)	0.8829	ns
TSH	µUL/mL	2.2 (1.6-3.0)	2.3 (1.8-3.35)	1.48 (1.35-1.71)	0.0730	ns
FT4	ng/dL	1.2 (1.0-1.3)	1.2 (1.05-1.28)	1.13 (1.07-1.21)	0.7506	ns
Ferritin	ng/mL	68.1 (43.7-81.9)	73.7 (44.9-120.0)	72.2 (41.2-101.8)	0.4829	ns
Vitamin D	ng/mL	21.7 (19.1-24.8)	20.5 (18.2-24.2)	20.6 (14.9-24.4)	0.7230	ns
CRP	mg/dL	0.25 (0.10-0.62)	0.20 (0.08-0.41)	0.46 (0.10-0.76)	0.5051	ns
TGF-β	pg/mL	4.58 (0.11-15.3)	579.6 (20.67-796.2)	521.8 (73.52-637.3)	**0.0001**	**a; b**
HO-1	pg/mL	47.9 (12.9-98.4)	22.0 (11.0-65.5)	30.32 (10.0-110.6)	0.5385	ns

Data represent medians and interquartile ranges. Data was analyzed using Kruskal-Wallis test with Dunn's multiple comparisons. P-values in bold font were statistically significant. *Ad hoc* comparisons with P-value <0.05: [a]No steatosis vs. Mild steatosis, [b]No steatosis vs. Moderate/Severe steatosis. ns: nonsignificant.

Figure S1. Overall profile of concentrations of biochemical parameters according to occurrence of hepatic steatosis and degree of obesity.

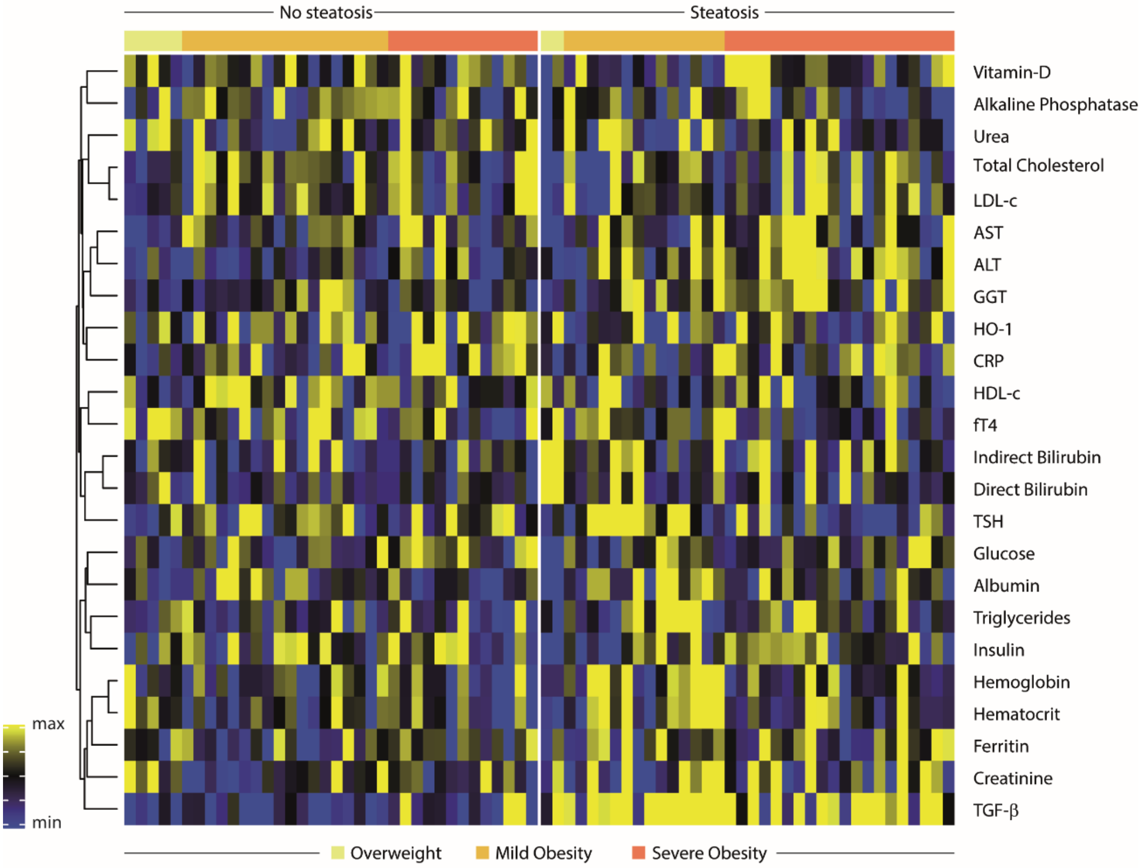

Values of indicated parameters were assessed in samples from patients without hepatic steatosis (n=36) and patients with hepatic steatosis (n=36), who were ordered according to degree of obesity. Data were Log10 transformed and z-score normalized. A hierarchical cluster analysis (Ward's method with 100X bootstrap) was employed to test whether there were differences in the overall expression profile of the biochemical parameters in the study population.

Figure S2. Correlations between values of BMI/age, ALT and TGF-β in the study population.

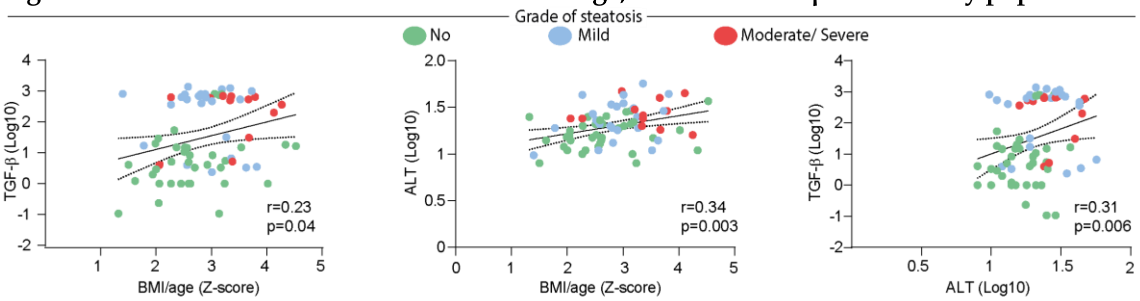

Data were analyzed using the Spearman correlation rank test. Lines represent linear curve fit with 95% confidence intervals.

Figure S3. Degree of biochemical perturbation of each biomarker according to grade of hepatic steatosis.

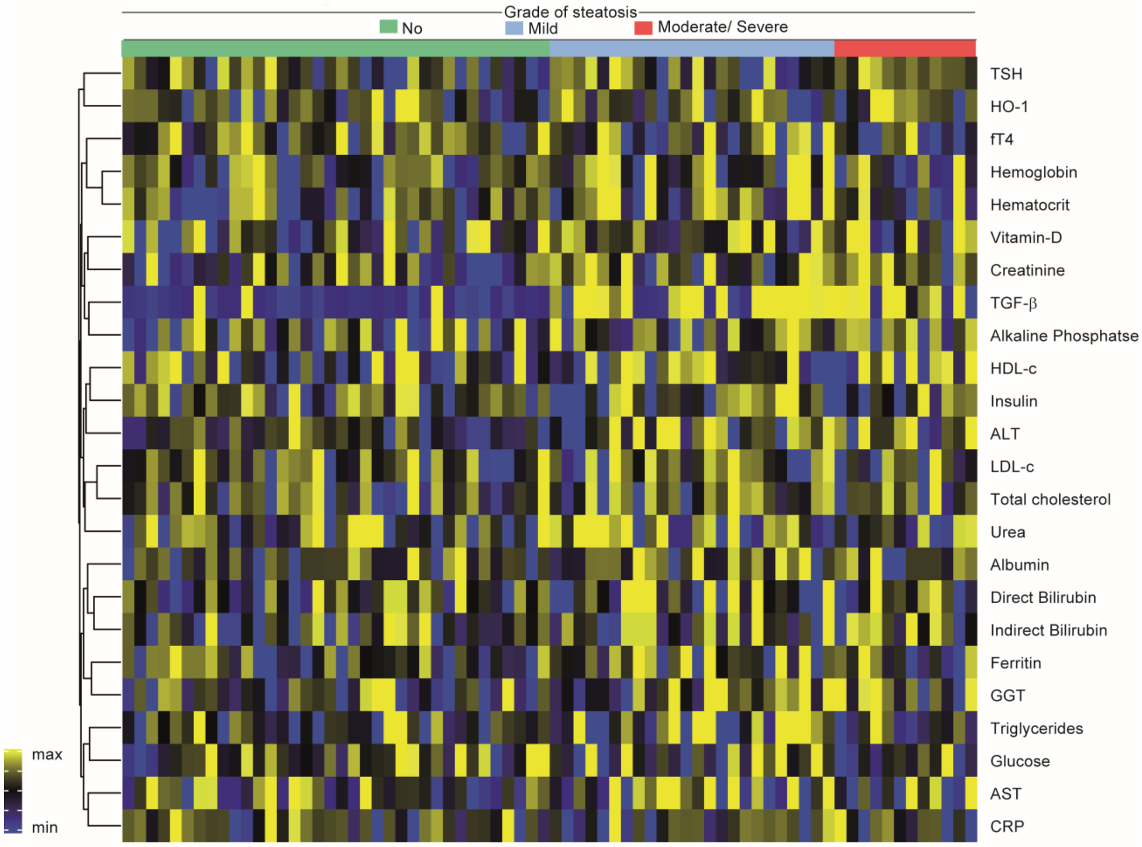

A one-way hierarchical cluster analysis (Ward's method with 100X bootstrap) using data on degree of biochemical perturbation calculated for each individual parameter was employed to visualize the profiles potentially associated degree of steatosis. To plot the heatmap, data were log10-transformed, and z-score normalized.

Figure S4. Degree of biochemical perturbation of each biomarker according to degree of obesity.

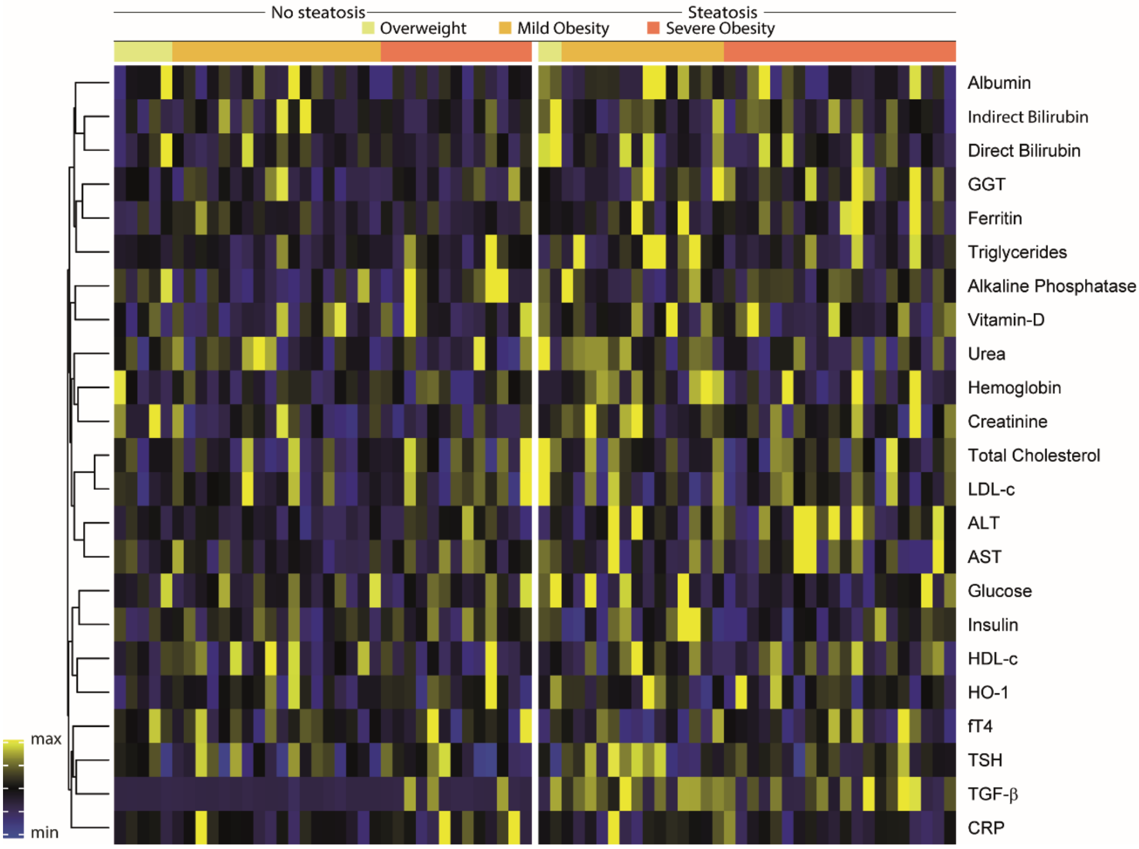

A one-way hierarchical cluster analysis (Ward's method with 100X bootstrap) using data on degree of biochemical perturbation calculated for each individual parameter was employed to visualize the profiles potentially associated degree of obesity. To plot the heatmap, data were log10-transformed, and z-score normalized.
